# New biomarkers exploration and nomogram construction of prognostic and immune-related adverse events of advanced non-small cell lung cancer patients receiving immune checkpoint inhibitors

**DOI:** 10.1186/s12931-023-02370-0

**Published:** 2023-02-27

**Authors:** Xuwen Lin, Xi Chen, Xiang Long, Chao Zeng, Zhihan Zhang, Weiyi Fang, Ping Xu

**Affiliations:** 1 Department of Pulmonary and Critical Care Medicine, Peking University Shenzhen Hospital, Shenzhen, 518034 Guangdong China; 2 Shantou University Medical College, Shantou, 515041 Guangdong People’s Republic of China; 3 Cancer Research Institute, School of Basic Medical Science, Southern Medical University, Guangzhou, 510515 Guangdong China; 4 Cancer Center, Integrated Hospital of Traditional Chinese Medicine, Southern Medical University, Guangzhou, 510315 China

**Keywords:** Immune, Adverse events, Lung cancer, Biomarkers, Nomogram

## Abstract

**Background:**

Immune checkpoint inhibitors (ICIs) are regarded as the most promising treatment for advanced-stage non-small cell lung cancer (aNSCLC). Unfortunately, there has been no unified accuracy biomarkers and systematic model specifically identified for prognostic and severe immune-related adverse events (irAEs). Our goal was to discover new biomarkers and develop a publicly accessible method of identifying patients who may maximize benefit from ICIs.

**Methods:**

This retrospective study enrolled 138 aNSCLC patients receiving ICIs treatment. Progression-free survival (PFS) and severe irAEs were end-points. Data of demographic features, severe irAEs, and peripheral blood inflammatory-nutritional and immune indices before and after 1 or 2 cycles of ICIs were collected. Independent factors were selected by least absolute shrinkage and selection operator (LASSO) combined with multivariate analysis, and incorporated into nomogram construction. Internal validation was performed by applying area under curve (AUC), calibration plots, and decision curve.

**Results:**

Three nomograms with great predictive accuracy and discriminatory power were constructed in this study. Among them, two nomograms based on combined inflammatory-nutritional biomarkers were constructed for PFS (1 year-PFS and 2 year-PFS) and severe irAEs respectively, and one nomogram was constructed for 1 year-PFS based on immune indices. ESCLL nomogram (based on ECOG PS, preSII, changeCAR, changeLYM and postLDH) was constructed to assess PFS (1-, 2-year-AUC = 0.893 [95% CI 0.837–0.950], 0.828 [95% CI 0.721–0.935]). AdNLA nomogram (based on age, change-dNLR, changeLMR and postALI) was constructed to predict the risk of severe irAEs (AUC = 0.762 [95% CI 0.670–0.854]). NKT-B nomogram (based on change-CD3+CD56+CD16+NKT-like cells and change-B cells) was constructed to assess PFS (1-year-AUC = 0.872 [95% CI 0.764–0.965]). Although immune indices could not be modeled for severe irAEs prediction due to limited data, we were the first to find CD3+CD56+CD16+NKT-like cells were not only correlated with PFS but also associated with severe irAEs, which have not been reported in the study of aNSCLC-ICIs. Furthermore, our study also discovered higher change-CD4+/CD8+ ratio was significantly associated with severe irAEs.

**Conclusions:**

These three new nomograms proceeded from non-invasive and straightforward peripheral blood data may be useful for decisions-making. CD3+CD56+CD16+NKT-like cells were first discovered to be an important biomarker for treatment and severe irAEs, and play a vital role in distinguishing the therapy response and serious toxicity of ICIs.

## Introduction

Lung cancer is one of the deadliest cancers globally [1]. Non-small cell lung cancer (NSCLC) causes 76% of lung cancer cases [2, 3]. Immune checkpoint inhibitors (ICIs), targeting programmed-cell death 1 (PD-1) and programmed-cell death ligand 1 (PD-L1) have transformed the treatment landscape of advanced-stage NSCLC (aNSCLC) [4]. However, only 15–20% experience durable response, others displaying primary or secondary resistance [5, 6]. Beyond that, ICIs may even cause severe immune-related adverse events (irAEs), which often come with the recommendation of discontinuing all further immunotherapy [7, 8]. PD-L1 in tissue is being explored as a predictive biomarker for outcomes of PD-L1/PD-1 treatment in NSCLC, while there are some conflicting data from the predictive biomarkers of response in terms of the level of PD-L1 expression. Many challenges remain in the clinical use of PD-L1, including different companion diagnostic assays, geographical heterogeneity, insufficient tissue for dynamic monitoring, and high levels of inter-assay variability in terms of both performance and cut-off points [9].

Recently, there has been a growing interest in the field of peripheral blood to identify non-invasive, cost-effective, easily accessible, and promising prognostic biomarkers to predict prognostic and toxicity of ICIs [10]. Consequently, many peripheral blood inflammatory-nutritional indices such as albumin, lactate dehydrogenase (LDH), neutrophil-to-lymphocyte ratio (NLR), derived NLR (dNLR), and C-reactive protein-albumin ratio (CAR) were found to link with metabolic homeostasis and affect the survival outcomes of ICIs [11–17]. Moreover, lymphocyte subsets, including CD3+, CD4+ and CD8+ T cell subsets, natural killer (NK) cells, and B cells, comprise a major population of tumor-infiltrating immune indices in circulating and are also of potential value for early judgment of clinical efficacy in ICIs [18–21]. However, their predictive values were verified in different studies and remained controversial. Until now, there has been no unified accuracy and comprehensive biomarkers and systematic construction model specifically identified for prognostic effect and severe toxicity.

The development of easy-to-use clinical tools able to identify ICIs benefit patients, resistant patients, and severe irAEs patients represents an unmet clinical need. Therefore, to predict therapeutic prognostic and early severe toxicity recognition for decision-making in the palliative treatment of aNSCLC patients receiving ICIs, the goal of the present study was to discover new predictive biomarkers and develop novel nomograms of prognostic and severe irAEs of aNSCLC patients receiving ICIs. The predictive biomarkers in these nomograms were based on a comprehensive assessment of clinical features, peripheral blood inflammatory-nutritional, and immune indices, which were more convenient and feasible for clinical acquisition.

## Methods

### Patients recruitment

**Fig. 1:**
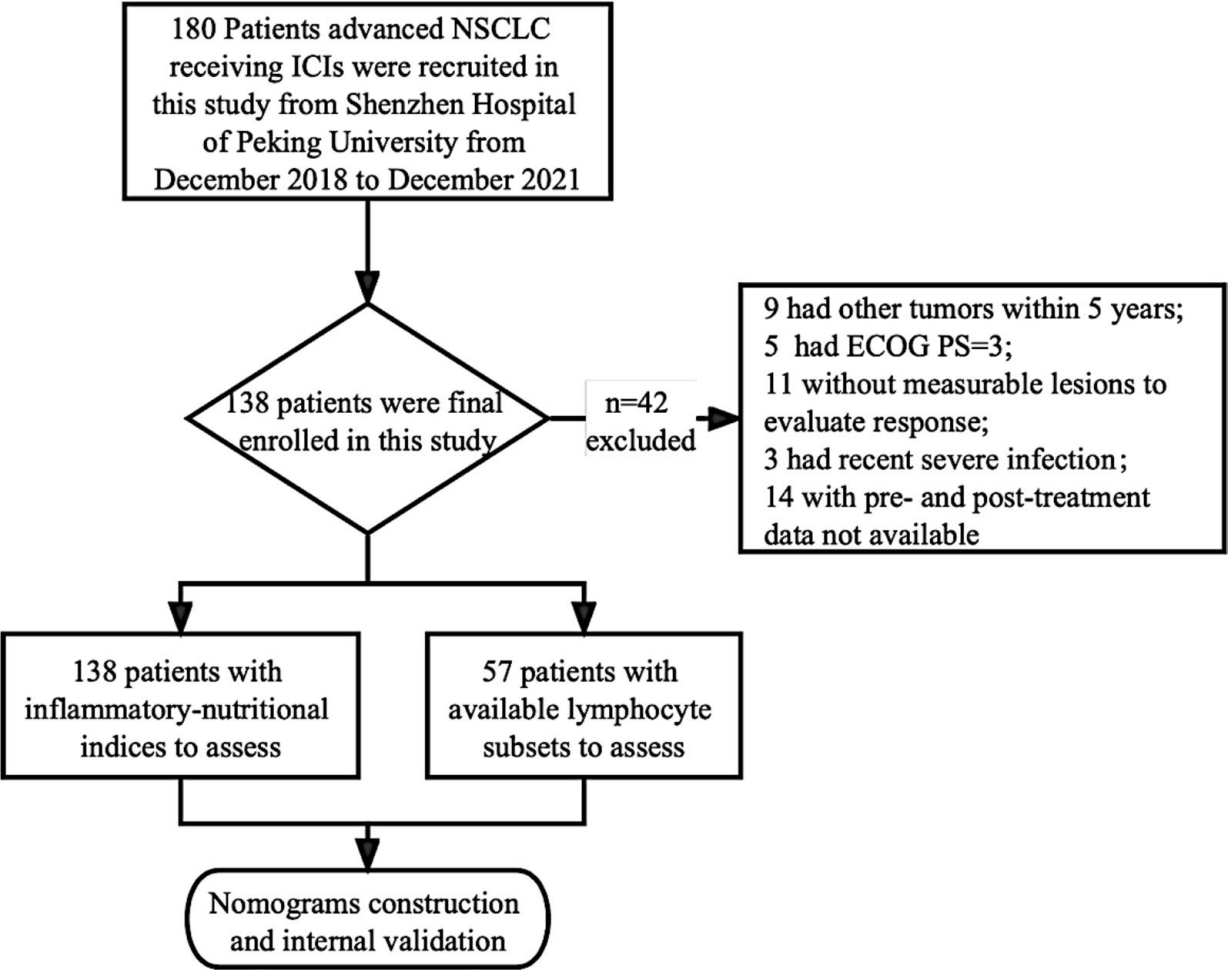
Flow chart of patient enrollment

In this study, a total of 180 advanced aNSCLC patients receiving ICIs treatment were initially recruited from Peking University Shenzhen Hospital from December 2018 to December 2021. The follow-up period ended on August 1st, 2022. Following the inclusion and exclusion criteria, as bellowed, 138 patients receiving ICIs (including Pembrolizumab, Nivolumab, Camrelizumab and Sintilimab) were enrolled in the final analysis (Fig. 1). The inclusion criteria were as follows: (i) above 18 years of age receiving ICIs treatment, or ICIs-based combination chemotherapy; (ii) pathologically or cytologically diagnosed clinical stage IIIB or stage IV NSCLC; (iii) Eastern Cooperative Oncology Group performance status scores (ECOG PS) between 0 and 2; (iv) no recent infection, trauma and not in the use of immune-regulatory drugs. The exclusion criteria were as follows: (i) patients with other types of tumors within 5 years; (ii) absence of measurable lesions to evaluate response according to immune-modified response evaluation criteria in solid tumors (imRECIST) criteria [22].

### Data collection

Data were collected at pre-treatment (within 7 days prior to ICIs, presented as “pre”) and post-treatment (after 1 or 2 cycles ICIs, presented as “post”), and the percentage change between the values (presented as “change = [post–pre]/pre”) was calculated as follows: (i) demographic features and clinical manifestations: gender, age, body mass index (BMI, kg/m^2^), ECOG PS, smoking history, comorbidities, treatment types, treatment lines, histology, TNM staging, driver gene mutation (EGFR, ALK, MET, HER-2, K-RAS, ROS-1, BRAF,TP53, PIK3CA), PD-L1 expression, and tumor mutation burden (TMB) when available; (ii) Peripheral blood inflammatory-nutritional indices: white blood cell count (WBC, E + 9/L), neutrophil count (NEU, E + 9/L), absolute lymphocyte count (LYM, E + 9/L), monocyte count (MONO, E + 9/L), hemoglobin (HB, g/L), albumin (Alb, g/L), LDH (U/L), C-reactive protein (CRP, mg/L), IL-6 (pg/mL), neutrophil–lymphocyte ratio (NLR), derived NLR (dNLR): NEU/(WBC − NEU), platelet-lymphocyte ratio (PLR), lymphocyte-to-monocyte ratio (LMR), prognostic nutritional index (PNI): Alb (g/L) + 5 × LYM; advanced lung cancer inflammation index (ALI): BMI × Alb (g/dL)/NLR, C-reactive protein-albumin ratio (CAR), system inflammation response index (SIRI): NEU × MONO/LYM, systemic immune inflammation index (SII): platelet count × NEU/LYM. (iii) Peripheral blood immune indices—lymphocyte subsets detected by flow cytometry assay: total T cells (CD3+ cells, Cells/μL), CD4+ T cells (Cells/μL), CD8+ T cells (Cells/μL), CD4+/CD8+ ratio (%), CD3+CD56+CD16+ Natural killer T (NKT)-like cells (Cells/μL), B cells (CD3−CD19+ cells, Cells/μL) and NK cells (CD3−CD16+CD56+ cells, Cells/μL).

### Follow-up and irAEs recognition

The follow-up on patients’ clinical outcomes was performed by referring to the clinic's attendance records and phone calls. According to imRECIST [22], those whose tumor growth increased at least 20% compared to pre-treatment were defined as progression diseases (PD), whereas decreased by 30% were defined as partial response (PR) or stable disease (SD). The primary endpoint of this study was progression-free survival (PFS), measured from treatment initiation to disease progression or last follow-up. The second endpoint was severe irAEs. Data of irAEs reported by physicians in every cycle of ICIs treatment was recorded. irAEs were typically referred to as a broad spectrum of manifestations involving different organ systems, including the skin, endocrine, pneumological, cardiovascular, gastrointestinal, hepatic, neurological, hematological, and other rare irAEs [23]. According to Common Terminology Criteria Adverse Events, “irAEs of any grade” were defined as occurring in any grade of one or more events; “severe irAEs” were defined as irAEs of grades 3–4 or irAEs leading to discontinuation of medication.

### Statistical analysis

The statistical analysis was performed using R studio (version 2022.02.0; https://www.R-project.org), SPSS (version 25.0) and X-tile software version 3.6.1 (Yale University). Missing data but no more than 10% missing were imputed with multiple imputations by the chained equations (MICE) approach. Categorical variables were expressed as n (%). Continuous data are presented as mean (± SD) or median (IQR, interquartile range). The best cut-off value of all variables for binary classification of PFS was recognized by X-tile. For the establishment of nomogram, firstly, we used the least absolute shrinkage and selection operator (LASSO) regression analysis to select the most useful prognostic factors. The optimal values of the penalty parameter λ were determined through tenfold cross-validation. Variables with zero regression coefficient are excluded while variables with nonzero are most strongly associated with the response. Secondly, variables with statistical significance (P < 0.05) in multivariate COX or logistic regression analysis were selected for constructing the nomogram. R package ‘rms’ was used for establishing and applying the nomogram. And each variable had a weighted score calculated by their respective coefficients, and the sum of the risk scores was associated with PFS or severe irAEs. The performance of nomogram was evaluated by area under (AUC) receiver operating characteristic curve (ROC), and calibration plots with bootstrapping (1000 resamples). Decision curve analysis (DCA) was used to assess whether patients could benefit from ICIs based on the nomogram (1000 resamples bootstrapping in logistic regression). Patients using nomograms for the prediction of PFS were split into low-risk and high-risk groups according to the optimal cut-off value of model risk score. Kaplan–Meier method and log-rank tests were used to assess differences in PFS between the predicted high-risk and low-risk groups. All p-values were set at P < 0.05 based on the two-tailed test.

## Results

### Patients’ clinical characteristics

The baseline characteristics of 138 aNSCLC patients with ICIs treatment were listed in Table 1. Among them, 44.9% were above 65 years of age, 81.9% were males, 64.5% were smokers, and 75.4% had an ECOG PS score < 2. In terms of clinical pathology, the proportion of squamous cell carcinoma (33.3%) was less than that of adenocarcinoma (58.0%), and more than half of patients (60.1%) had distant metastasis before ICIs treatment onset. 49.3% were evaluated in PD-L1 expression and 9.4% are detected as negative. 23.9% were evaluated in TMB testing and 10.9% demonstrated TMB-High expression. What’s more, there were 15.9% of the patients showed driver gene mutations in our study, and 7.2% were EGFR-sensitive mutation positive. In addition, 37.7% received ICIs monotherapy and 21.7% had received three or more prior lines of therapy.

**Table 1:** Clinical characteristics of 138 aNSCLC patients receiving ICIs treatment

Clinical characteristics	Level	Overall (n = 138)
Gender (%)	Female	25 (18.1)
	Male	113 (81.9)
Age (%)	< 65	76 (55.1)
	≥ 65	62 (44.9)
BMI (%)	< 24	38 (27.5)
	≥ 24 < 28	73 (52.9)
	≥ 28	27 (19.6)
Smoking (%)	No	49 (35.5)
	Yes	89 (64.5)
ECOG PS (%)	< 2	104 (75.4)
	≥ 2	34 (24.6)
Distant metastasis (%)	No	55 (39.9)
	Yes	83 (60.1)
Histology (%)	Adenocarcinoma	80 (58.0)
	Squamous	46 (33.3)
	Others	12 (8.7)
PD-L1 (%)	< 1%	13 (9.4)
	≥ 1%, < 50%	28 (20.3)
	≥ 50%	27 (19.6)
	Unknown	70 (50.7)
TMB (%)	TMB-high	15 (10.9)
	TMB-low	18 (13.0)
	Unknown	105 (76.1)
Commodities (%)	Hypertension	33 (23.9)
	Diabetes	13 (9.4)
	Coronary heart disease	12 (8.7)
	COPD	10 (7.2)
	Bronchiectasis	14 (10.1)
	Latent tuberculosis	6 (4.3)
	Hepatitis B	11 (8.0)
	Thyroid nodules	16 (11.6)
Mutation types (%)	BRAF	1 (0.7)
	EGFR	7 (5.1)
	EGFR + KARS	2 (1.4)
	EGFR + TP53	1 (0.7)
	HER2	1 (0.7)
	HER2 + PIK3CA	1 (0.7)
	KRAS	2 (1.4)
	MET	1 (0.7)
	PIK3CA	1 (0.7)
	TP53	5 (3.6)
	No	116 (84.1)
Treatment types (%)	Chemoimmunotherapy	86 (62.3)
	Immunotherapy	52 (37.7)
Treatment lines (%)	First line	83 (60.2)
	Second line	25 (18.1)
	≥ 3 lines	30 (21.7)
irAEs of any grade (%)	Non-irAEs	63 (45.7)
	irAEs	75 (54.3)
severe irAEs (%)	Non-Grade3/4	99 (71.7)
	Grade3/4	39 (28.3)
irAEs number (%)	0	65 (47.1)
	1	47 (34.1)
	2	18 (13.0)
	3	5 (3.6)
	≥ 4	3 (2.2)
irAEs types (%)	Thyroid disorders	22 (15.9)
	Skin rash	23 (16.7)
	Diarrhea	13 (9.4)
	Hepatitis	19 (13.8)
	Renal	7 (5.1)
	Pneumonitis	19 (13.8)
	Cardiovascular	8 (5.8)
	Neurotoxicity	1 (0.7)
	Thrombocytopenia	2 (1.4)
PFS (month, median [IQR])		11.0 (3.55–21.23)
PFS status (%)	Non-recurrence	44 (37.6)
	Recurrence	73 (62.4)
Best overall response (%)	PD + SD	51 (43.6)
	PR	66 (56.4)

*aNSCLC* advanced-stage non-small cell lung cancer, *ICIs* immune checkpoint inhibitors, *irAEs* immune-related adverse events, *TMB* tumor mutational burden, *ECOG PS* Eastern Cooperative Oncology Group performance status scores, *COPD* chronic obstructive pulmonary disease, *PFS* progression-free survival, *PD* progression diseases, *PR* partial response, *SD* stable disease

During follow-up, 117 patients were available for survival outcome, while the remaining 21 discontinued treatment due to severe irAEs. The median PFS was 11.0 months (IQR 3.55–21.23). In terms of irAEs in 138 aNSCLC patients, approximately half of patients (75, 54.3%) experienced at least one irAE and a smaller number of them (39, 28.3%) experienced severe irAEs reported in total. The most common irAEs involved skin rash (23, 16.7%), thyroid disorders (22, 15.9%), pneumonitis (19, 13.8%) and hepatitis (19, 13.8%), followed by the diarrhea (13, 9.4%), cardiovascular (8, 5.8%), renal (7, 5.1%), neurotoxicity (1, 0.7%), thrombocytopenia (2, 1.4%).

### New biomarkers exploration and nomograms construction

**Fig. 2:**
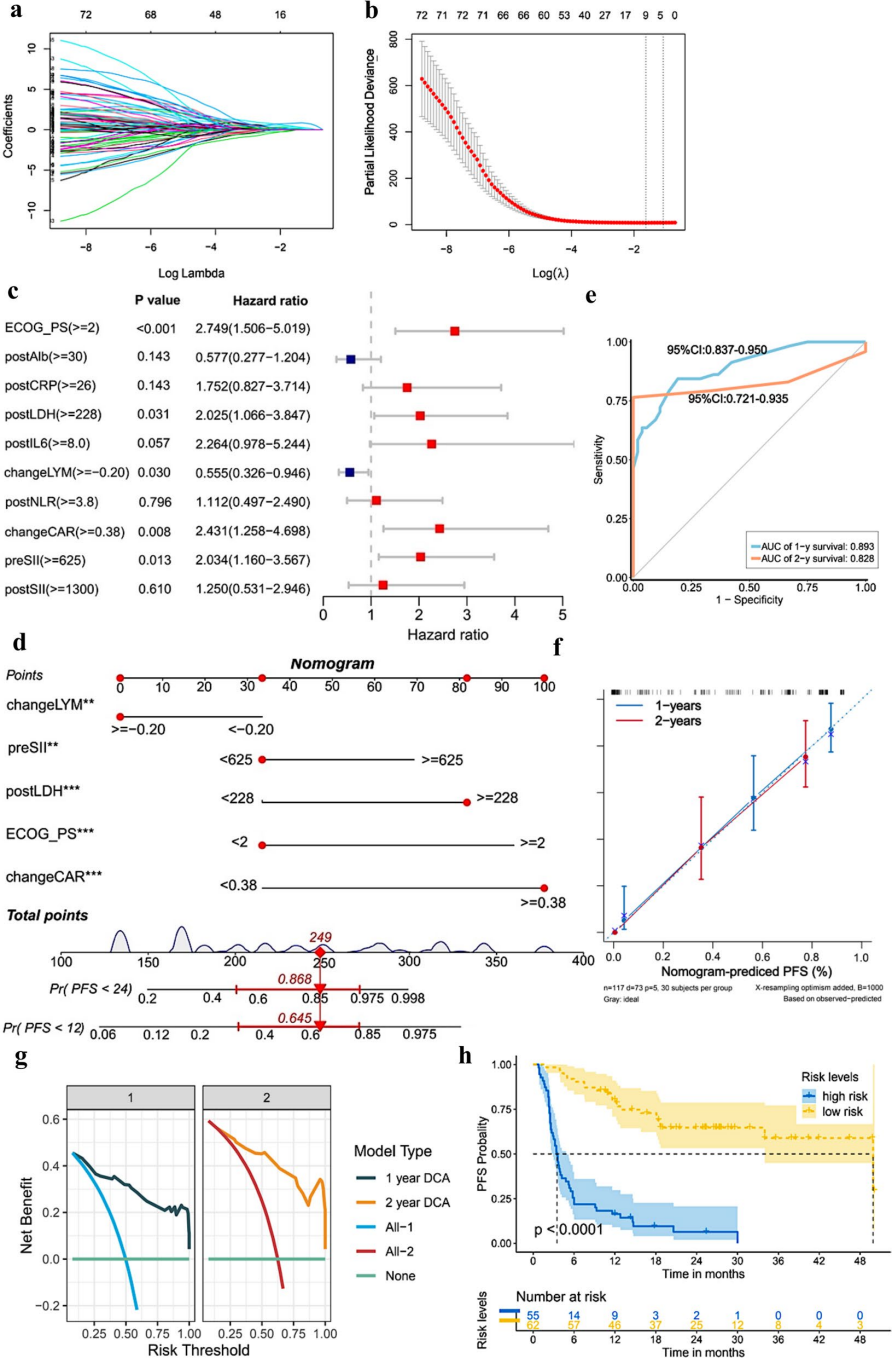
Factors selection and nomogram construction based on peripheral-blood inflammatory-nutritional indices of PFS. **a** The LASSO coefficient profiles of candidate variables; **b** the optimal tuning parameter lambda λ in LASSO analysis selected with tenfold cross-validation. Dotted vertical line is set at the nonzero coefficients selected via tenfold cross-validation. The first vertical line equals the minimum error. The second vertical line shows cross-validated error within one standard error of the minimum; **c** forest plot of multivariate analysis of PFS. **d** “ESCLL” nomogram (based on ECOG PS, preSII, changeCAR, changeLYM and postLDH) was established to assess 1-year and 2-year PFS; Each factor corresponds to a specific point by drawing a vertical line from that variable to the points axis. After sum of the scores for each variable located on the Total Points axis. Finally, the sum represents the probability of 1-, 2-year survival by drawing straight down to the survival axis. (For example, an aNSCLC patient with changeLYM ≥ − 0.20, preSII < 625, postLDH ≥ 228, ECOG PS < 2 and changeCAR ≥ 0.38, the total score will be given by 249, corresponding to 1- and 2-year risks of progression of 0.645 and 0.868, respectively. The patient will accordingly have approximately 35.5% and 13.2% survival probabilities at 1 and 2 years, respectively) *P < 0.05; **P < 0.01; ***P < 0.001. **e** The ROC curve of the nomogram for 1-year, 2-year PFS; **f** calibration curves of the nomogram for 1-year, 2-year PFS; **g** decision curve analysis of the nomogram for 1-year, 2-year PFS; **h** Kaplan–Meier curves depicting PFS according to risk levels (cutpoint value: 1.37). Significant difference in survival of patients was observed between high and low risk score. ECOG PS, Eastern Cooperative Oncology Group Performance Status scores; preSII, pre-treatment systemic immune inflammation index; postLDH, post-treatment lactate dehydrogenase; changeLYM, change of absolute lymphocyte count; changeCAR, change of C-reactive protein-albumin ratio; ROC, receiver operator characteristic

**Fig. 3:**
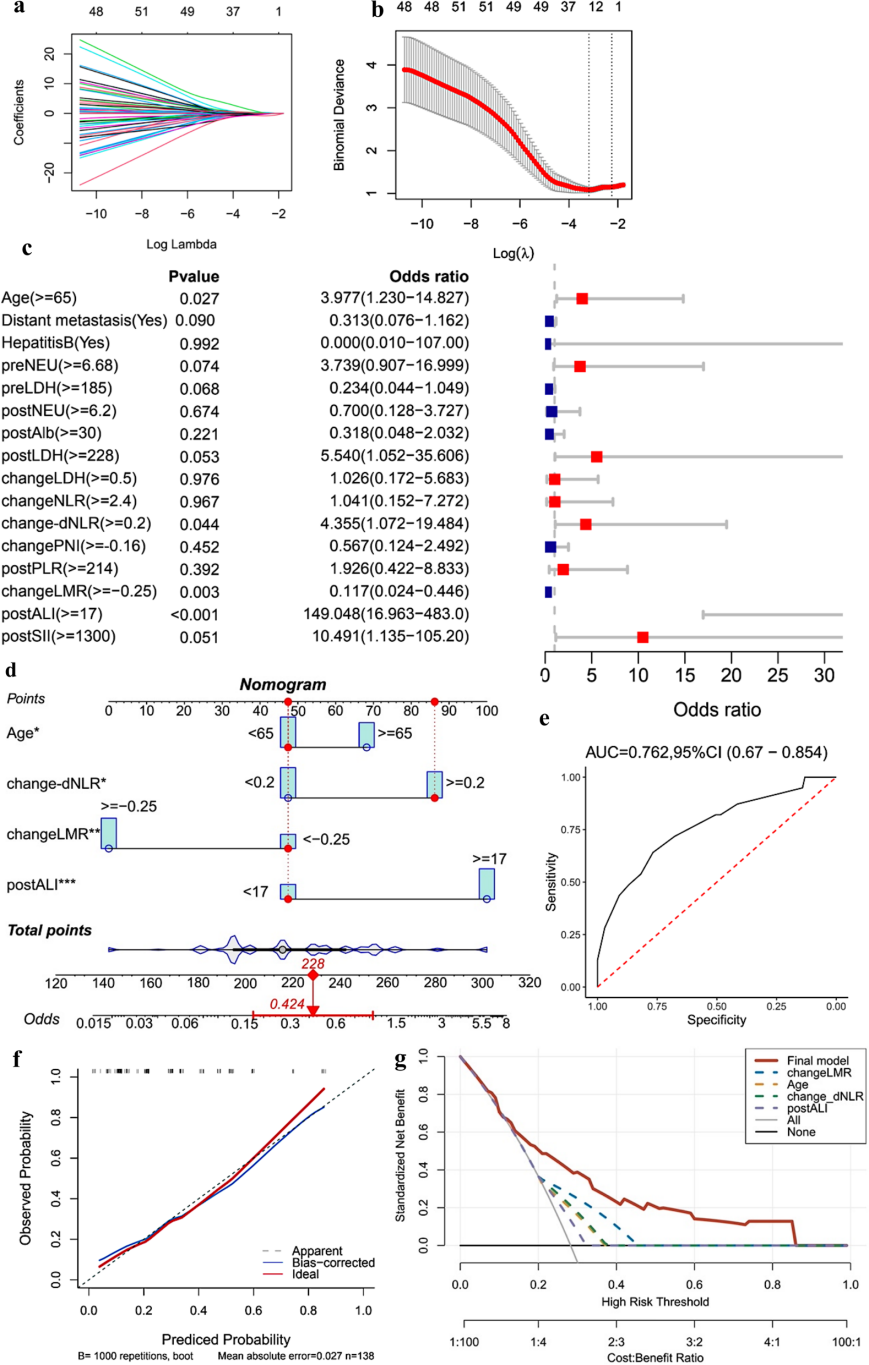
Factors selection and nomogram construction based on peripheral-blood inflammatory-nutritional indices of severe irAEs. **a** The LASSO coefficient profiles of the candidate variables; **b** the optimal tuning parameter (lambda, λ) in the LASSO analysis selected with tenfold cross-validation via minimum criteria; **c** forest plot of multivariate analysis independent prognostic analysis of severe irAEs; **d** the “AdNLA” nomogram (based on age, change-dNLR, changeLMR and postALI) were constructed for severe irAEs prediction of aNSCLC patients receiving ICIs; **e** the ROC curve of the nomogram for severe irAEs; **f** calibration curves of the nomogram for severe irAEs; **g** decision curves analysis of the nomogram for severe irAEs. *irAEs* immune-related adverse events, *change-dNLR* change of derived-neutrophil–lymphocyte ratio, *changeLMR* change of lymphocyte-to-monocyte ratio, *postALI* post-treatment advanced lung cancer inflammation index

**Fig. 4:**
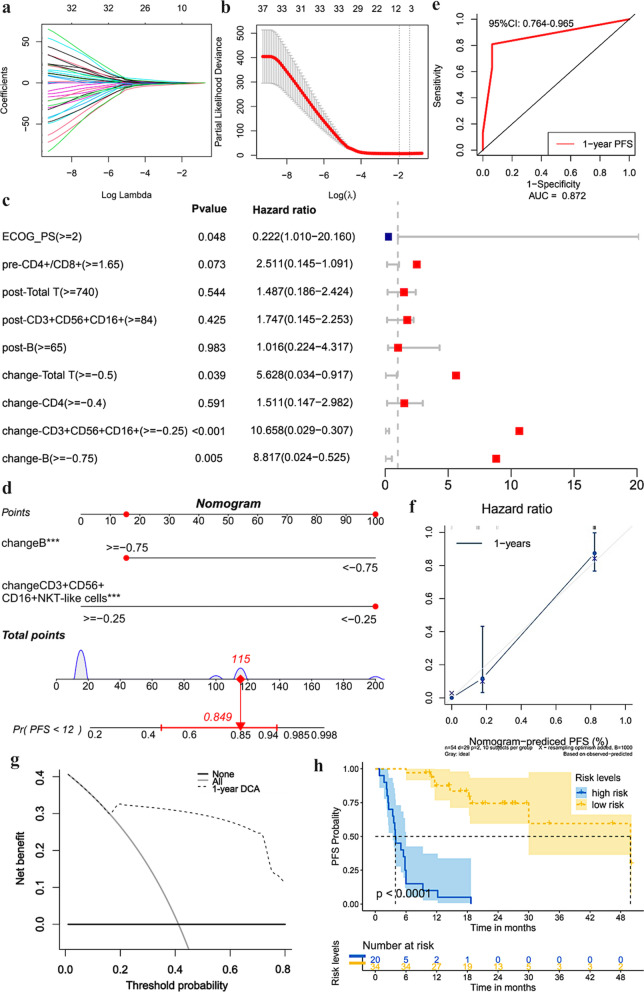
Factors selection and nomogram construction based on peripheral blood immune indices of PFS. **a** The LASSO coefficient profiles of the candidate variables; **b** the optimal tuning λ in the LASSO analysis selected with tenfold cross-validation via minimum criteria; **c** forest plot of multivariate analysis independent prognostic analysis of PFS. **d** “NKT-B” nomogram (based on change-CD3+CD56+CD16+NKT-like cells and change-B cells) was established to assess 1-year PFS of aNSCLC patients receiving ICIs; **e** the ROC curve of the nomogram for 1-year PFS; **f** calibration curves of the nomogram for 1-year PFS; **g** decision curve analysis of the nomogram for 1-year PFS; **h** Kaplan–Meier curves depicting PFS according to risk levels (cut-point value: − 4.1868)

In this study, we constructed three nomograms for PFS and severe irAEs prediction (Figs. 2, 3, 4). Among them, two nomograms based on combined inflammatory-nutritional biomarkers were constructed for PFS (1y-PFS and 2y-PFS) and severe irAEs, and one nomogram was constructed for 1y-PFS based on immune indices. These nomograms were as follows: (i) ESCLL nomogram (based on ECOG PS, preSII, changeCAR, changeLYM and postLDH) was constructed to assess 1-, 2-year PFS probability. (ii) AdNLA nomogram (based on age, change-dNLR, changeLMR and postALI) was constructed to predict severe irAEs. (iii) NKT-B nomogram (based on change-CD3+CD56+CD16+NKT-like cells and change-B cells) was constructed to assess 1-year PFS probability.

#### “ESCLL” nomogram based on inflammatory-nutritional indices for PFS assessment

As shown in Fig. 2a–c, taking PFS as dependent variables, 10 variables (ECOG PS, postAlb, postCRP, postLDH, postIL6, changeLYM, postNLR, changeCAR, preSII and postSII) based on clinical features and inflammatory-nutritional indices with non-zero coefficient were obtained from LASSO regression and were further incorporated into the Cox multivariate analysis. Multivariate results showed that ECOG PS ≥ 2 (HR 2.749; 95% CI 1.506–5.019; P < 0.001), preSII ≥ 625 (HR 2.034; 95% CI 1.160–3.567; P = 0.013), changeCAR ≥ 0.38 (HR 2.431; 95% CI 1.258–4.698; P = 0.008), changeLYM ≥ − 0.20 (HR 0.555; 95% CI 0.326–0.946; P = 0.030) and postLDH ≥ 228 (HR 2.025; 95% CI 1.066–3.847; P = 0.031) were independent predictors affecting the prognosis of PFS in aNSCLC patients receiving ICIs. We further developed a model that includes 5 independent predictors—ESCLL—and displayed it as a nomogram to predict the 1- and 2-year PFS (1y-PFS, 2y-PFS) probabilities (Fig. 2d). A subsequent ROC analysis revealed that our prognostic nomogram had favorable discrimination, with 1- and 2-year AUC of 0.893 (95% CI 0.837–0.950) and 0.828 (95% CI 0.721–0.935), respectively (Fig. 2e). The calibration plot revealed that the 1- and 2-year survival probabilities predicted by ESCLL nomogram had an excellent agreement with the actual observations (Fig. 2f). The DCA curve demonstrated that ESCLL had a net benefit for 1- and 2-year PFS across threshold probabilities (Fig. 2g). The prognostic model risk score for each patient was computed according to coefficient generated from the COX regression: the model risk score formula = 1.3199 × ECOG PS + 0.7961 × preSII + 1.4788 × changeCAR − 0.7447 × changeLYM + 1.0738 × postLDH. All patients were then stratified into high-risk and low-risk groups using the optimal cutoff of ESCLL nomogram risk scores (cutoff = 1.37, P < 0.001). The Kaplan–Meier survival curve showed patients in the high-risk group exhibited a significantly poorer PFS (P < 0.001) (Fig. 2h).

#### “AdNLA” nomogram based on inflammatory-nutritional indices for severe irAEs prediction

Combined with the results from LASSO and multivariate logistic analysis, we confirmed 4 independent indicators, including age ≥ 65 (OR 3.977; 95% CI 1.230–14.827; P = 0.027), change-dNLR ≥ 0.2(OR 4.355; 95% CI 1.072–19.484; P = 0.044), changeLMR ≥ − 0.25(OR 0.117; 95% CI 0.024–0.446; P = 0.003) and postALI ≥ 17(OR 149.048; 95% CI 16.963–483.0; P < 0.001), for predicting the risk of severe irAEs (Fig. 3a–c). AdNLA nomogram based on the above predictors was constructed (Fig. 3d), exhibiting good model discrimination (AUC = 0.762, 95% CI 0.670–0.854) and calibration ability (Fig. 3e, f). The model risk score formula = 0.7480 × age + 1.3961 × change-dNLR − 1.7077 × changeLMR + 1.8928 × postALI. In addition, DCA showed across threshold probability, using AdNLA to predict severe irAEs incidence risk could be beneficial (Fig. 3g).

#### “NKT-B” nomogram based on immune indices for PFS assessment

57 patients had complete lymphocyte subsets data, of which 3 patients discontinued treatment due to severe irAEs. As shown in Fig. 4a–c, 9 variables (ECOG PS, pre-CD4+/CD8+ ratio, post-Total T cells, post-CD3+CD56+CD16+NKT-like cells, post-B cells, change-Total T cells, change-CD4+ T cells, change-CD3+CD56+CD16+NKT-like cells, and change-B cells) based on clinical features and immune indices with non-zero coefficient were obtained from LASSO regression and were further incorporated into the Cox multivariate analysis. Multivariate analysis showed that ECOG PS ≥ 2 (OR 0.222; 95% CI 1.010–20.160; P = 0.048), change-Total T cells ≥ − 0.5 (OR 5.628; 95% CI 0.034–0.917; P = 0.039), change-CD3+CD56+CD16+NKT-like cells ≥ − 0.25 (OR 10.658; 95% CI 0.029–0.307; P < 0.001) and change-B cells ≥ − 0.75 (OR 8.817; 95% CI 0.024–0.525; P = 0.005) were independent predictors associated with poor PFS. Notably, change-CD3+CD56+CD16+NKT-like cells had the highest OR, followed by change-B cells. Thus, we further selected change-CD3+CD56+CD16+NKT-like cells and change-B cells incorporated into new nomogram construction (namely NKT-B) to predict PFS probabilities (Fig. 4d). The model risk score formula = − 2.2680 × change-CD3+CD56+CD16+NKT-like cells − 1.9188 × change-B cells. NKT-B nomogram demonstrated great predictive accuracy (1-year-AUC = 0.872, 95% CI 0.764–0.965) and goodness of fit in the calibration plot (Fig. 4e, f). Additionally, DCA demonstrated that NKT-B had the highest net benefit for 1y-PFS across threshold probabilities (Fig. 4g). Kaplan–Meier survival curves showed that patients with high-risk scores exhibited significantly poor 1y-PFS (cutoff = − 4.1868, P < 0.001) (Fig. 4h).

#### Correlation of lymphocyte subsets with severe irAEs in aNSCLC with ICIs treatment

**Fig. 5:**
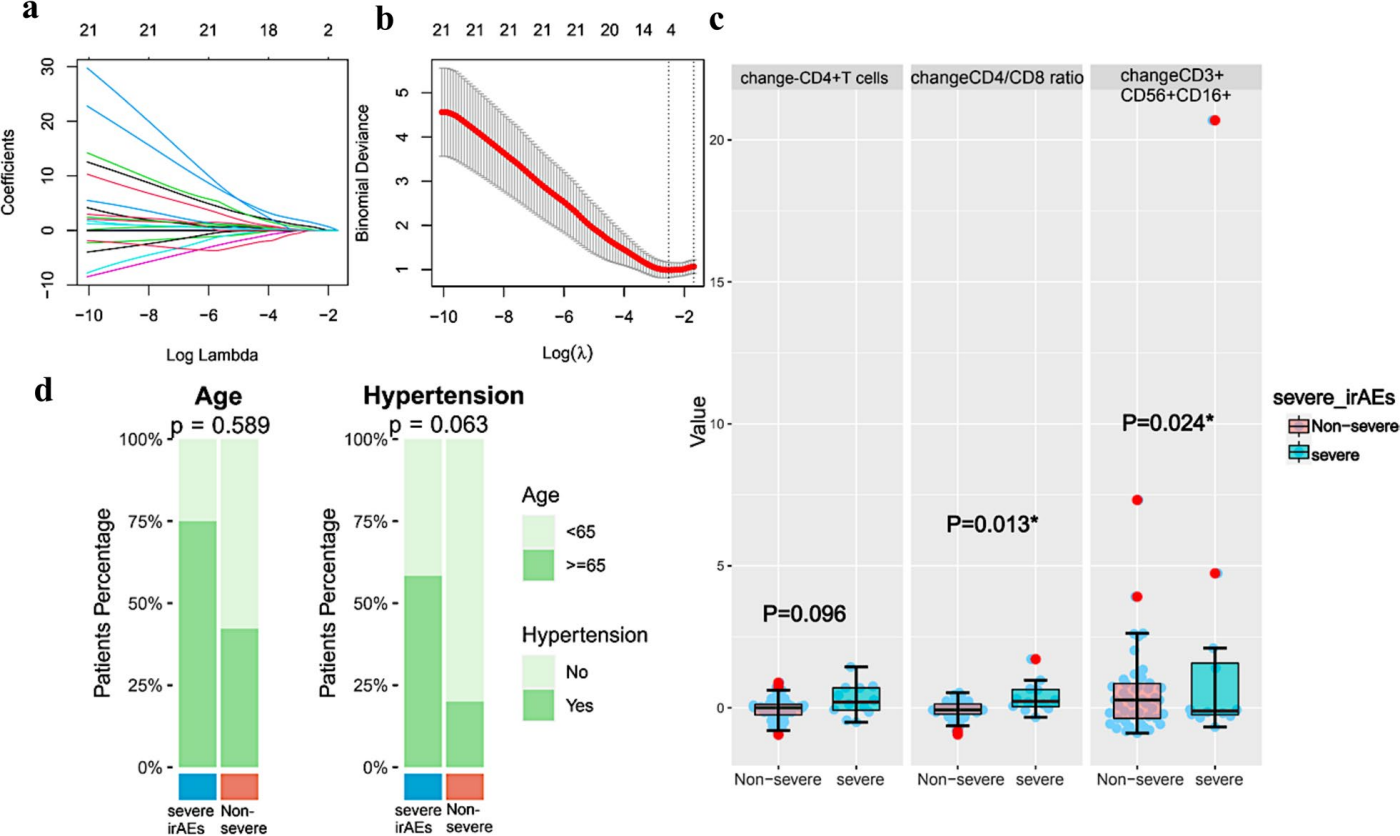
Factors selection and correlation analysis based on peripheral-blood immune indices of severe irAEs. **a** The LASSO coefficient profiles of the candidate variables; **b** the optimal tuning λ in the LASSO analysis selected with tenfold cross-validation via minimum criteria; **c** the development of severe irAEs corresponds to change-CD4+ T cells, change-CD4+/CD8+ ratio and change-CD3+CD56+CD16+NKT-like cells; **d** the development of severe irAEs corresponds to age and hypertension

Since our sample size was limited in lymphocyte subsets detection, we chose to use those immune indices for association analysis instead of nomogram construction of severe irAEs. Variables retained by LASSO regression were shown in Fig. 5a, b. Multivariate analysis confirmed that changeCD3+CD56+CD16+NKT-like cells (P = 0.024) and changeCD4+/CD8+ ratio (P = 0.013) significantly were correlated with incidence of severe irAEs (Fig. 5c, d).

## Discussion

Identification of the biomarker set with maximal predictivity that is the easiest to apply in clinical practice to foresee ICIs benefit and serious toxicity is deeply needed in aNSCLC. While tissue PD-L1 expression is the most heavily studied biomarker, its definite role as a predictor of response to ICIs is more debated and problematic in geographical heterogeneity and insufficient tissue obtaining [24, 25]. As the noninvasive, low-cost, and reproducible clinical item, peripheral blood was used here as a source of common players of the inflammatory response and immunophenotypes critically implicated in PD-1/PD-L1 pathway [26]. Herein, we successfully established three new nomograms that demonstrate great predictive accuracy, including ESCLL nomograms for PFS (1y-PFS and 2y-PFS) and AdNLA nomograms for severe irAEs prediction respectively, based on integrated peripheral blood inflammatory-nutritional indices; in addition, NKT-B nomogram based on peripheral blood immune indices for 1y-PFS prediction of aNSCLC patients receiving ICIs. These noninvasive nomograms can help clinicians accurately predict outcomes, and identify those more likely to suffer toxicity from ICIs. Moreover, it was also notable that CD3+CD56+CD16+NKT-like cells, as special lymphocyte subset, could be a new potential biomarker of prognostic and severe irAEs. To our knowledge, that has not been reported in the study of aNSCLC ICIs.

In this study, it is noteworthy that higher postLDH and changeCAR were consistent predictors of PFS, which go beyond previous reports showing that LDH and CAR after ICIs treatment but not at baseline could be associated with poor prognostic [27–29]. Moreover, we also observed that poor ECOG PS, low changeLYM and high preSII were associated with worse PFS, which ties well with prior studies [30–33]. Another further novel finding was higher change-dNLR, postALI, and lower change-LMR could increase the risk of severe irAEs, giving new light on that early ICIs treatment could trigger circulating inflammation status and then even induce severe toxicity. Our results underscore the importance of the inflammation and nutrition status for aNSCLC patients with ICIs treatment [26]. However, most prior prognostic indices or models did not have a comprehensive analysis of peripheral inflammatory indices and may be affected by tumor-independent variables, such as infections or co-morbidities, partly limiting their clinical significance. Also of note, we further attempted to construct ESCLL nomogram incorporating ECOG PS, preSII, changeCAR, changeLYM and postLDH for 1y-PFS and 2y-PFS prediction. Intriguingly, it performs well, giving clearly better results than that of individuals and categorizing patients into high- and low-risk groups. Another key strength of our study was that AdNLA nomogram was developed, encompassing age, change-dNLR, changeLMR and postALI, and was able to early identify patients with severe irAEs. The power of this integrated model overcame that of individual scores. Our finding supports the notion that multiparametric strategies may improve our ability to anticipate the clinical response to ICIs and severe irAEs-specific early identification.

Moreover, considering that anti-tumor immunity is not only affected by inflammatory response but also in addition to facilitating activation of immune effector cells, we further screened the circulating lymphocyte subsets combined with clinical factors to provide immune profiles more representative of the multifaceted aspects of cancer immunity. Most notably, our observation is rather unique as it is, to our knowledge, the first to discover that change-CD3+CD56+CD16+NKT-like cells could be a new biomarker that was not only associated with favorable PFS but also a key discriminative feature of a group of patients with severe irAEs. Most prior studies have examined the immune profile of CD3+, CD4+, CD8+ T cells with yielded controversial results [34–38], but the importance of NKT-like cells was not investigated in ICIs-treated aNSCLC. Actually, NKT-like cells can express the surface markers of T cells (CD3+) and NK cells (CD56+ additional with CD16+) [39, 40]. Although NKT-like cells are poorly known, previous studies have proposed that the CD3+CD56+NKT-like cells may be a crucial effector for the antitumor activities in surgery-treated colon carcinoma, radiotherapy-treated gastric cancer, and ICIs-treated hepatocellular carcinoma patients [41, 42]. It was speculated that ICIs may trigger CD3+CD56+CD16+NKT-like cells to help screen benefit patient groups, however, this effect would be likely to indicate responses against self-antigens, which may lead to severe toxicity. What is also worth noticing is that high change-B cells group was linked to better PFS. Xia et al. [21] have reported that a high percentage of B cells before ICIs treatment positively impacted PFS in aNSCLC. To be noticed, our study not only highlighted the dynamic change of B cells but also focused on exploring the predictive value of absolute count. Moreover, it is shown that higher early change of CD4+/CD8+ ratio was significantly linked to severe irAEs, however, to our knowledge, has not been reported in the study of aNSCLC ICIs. Furthermore, we constructed a new immune-based nomogram NKT-B, which incorporated change-CD3+CD56+CD16+NKT-like cells and change-B cells to accurately predict 1y-PFS. We demonstrated that NKT-B outperformed each of the individual features alone and well in performance. As another noninvasive model previously published in CELL research, DIREct-On did not include peripheral blood inflammatory indices but pre-treatment CD8+ T cells and blood TMB, and early ctDNA change, which was associated with PFS in a retrospective study on 99 ICIs-treated aNSCLC patients [43]. It is particularly worth mentioning here that even though we did not replicate DIREct-On model, our results still suggested excellent performance (DIREct-On: AUC = 0.93; NKT-B: 0.872) and exploring new biomarkers, although this should be validated on a larger cohort.

Some limitations of the study should be noted. Firstly, this study is restricted to a one-center, retrospective, internal validated perspective. Future work might consider extending this study to a larger scale to externally validate. Secondly, due to the limited data on lymphocyte subsets, immune indices failed to be modeled for severe irAEs prediction and explored in conjunction with inflammatory-nutritional indices. Besides, tissue biopsy is not routinely performed in advanced patients, and thus PD-L1 expression has only been evaluated in small patient cohorts. Future studies could take into account additional data to present a more comprehensive and representative work. Thirdly, due to the time limitations, the overall survival was not reached which is a potentially important aspect that would add greater depth to the study.

## Conclusions

The three brand-new nomograms derived from peripheral blood inflammatory-nutritional and immune indices, and clinical factors have utility for predicting response and serious toxicity to ICIs in aNSCLC. Given that the difficulty to date in developing robust predictive biomarkers for immunotherapy, noninvasive early identification proceeded from low-cost and straightforward peripheral blood data of response and toxicity assessment could help fill the unmet need of improving personalization. Notably, CD3+CD56+CD16+NKT-like cells could be a new biomarker that plays a vital role in distinguishing the survival and serious toxicity of ICIs. These preliminary results represent a significant step forward but warrant further research on a larger cohort.

## Data Availability

The data generated in this study may be available upon reasonable request from the corresponding author.

## Abbreviations

ICIs: Immune checkpoint inhibitors
aNSCLC: Advanced-stage non-small cell lung cancer
PD-1: Programmed-cell death 1
PD-L1: Programmed-cell death ligand 1
irAEs: Immune-related adverse events
LASSO: Least absolute shrinkage and selection operator
TMB: Tumor mutational burden
ECOG PS: Eastern Cooperative Oncology Group performance status scores
COPD: Chronic obstructive pulmonary disease
PFS: Progression-free survival
PD: Progression diseases
PR: Partial response
SD: Stable disease
imRECIST: Immune-modified response evaluation criteria in solid tumors
WBC: White blood cell count
NEU: Neutrophil count
LYM: Absolute lymphocyte count
MONO: Monocyte count
HB: Hemoglobin
Alb: Albumin
LDH: Lactate dehydrogenase
CRP: C-reactive protein
IL-6: Interleukin-6
NLR: Neutrophil–lymphocyte ratio
dNLR: Derived neutrophil–lymphocyte ratio
PLR: Platelet-lymphocyte ratio
LMR: Lymphocyte-to-monocyte ratio
PNI: Prognostic nutritional index
ALI: Advanced lung cancer inflammation index
CAR: C-reactive protein-albumin ratio
SIRI: System inflammation response index
SII: Systemic immune inflammation index
NK cells: Natural killer cells
NKT: Natural killer T
AUC: Area under curve
ROC: Receiver operating characteristic curve
DCA: Decision curve analysis
MICE: Multiple imputations by the chained equations
IQR: Interquartile range
